# Dopamine D4 receptor polymorphism and sex interact to predict children’s affective knowledge

**DOI:** 10.3389/fpsyg.2015.00846

**Published:** 2015-06-23

**Authors:** Sharon Ben-Israel, Florina Uzefovsky, Richard P. Ebstein, Ariel Knafo-Noam

**Affiliations:** ^1^Department of Psychology, The Hebrew University of JerusalemJerusalem, Israel; ^2^Department of Psychology, Academic College of Tel Aviv-YaffoTel Aviv, Israel; ^3^Autism Research Centre, Department of Psychiatry, University of CambridgeCambridge, UK; ^4^Department of Psychology, National University of SingaporeSingapore, Singapore

**Keywords:** dopamine, *DRD4*, cognitive empathy, affective perspective taking, gender, affective knowledge

## Abstract

Affective knowledge, the ability to understand others’ emotional states, is considered to be a fundamental part in efficient social interaction. Affective knowledge can be seen as related to cognitive empathy, and in the framework of theory of mind (ToM) as affective ToM. Previous studies found that cognitive empathy and ToM are heritable, yet little is known regarding the specific genes involved in individual variability in affective knowledge. Investigating the genetic basis of affective knowledge is important for understanding brain mechanisms underlying socio-cognitive abilities. The 7-repeat (7R) allele within the third exon of the dopamine D4 receptor gene (*DRD4-III*) has been a focus of interest, due to accumulated knowledge regarding its relevance to individual differences in social behavior. A recent study suggests that an interaction between the *DRD4-III* polymorphism and sex is associated with cognitive empathy among adults. We aimed to examine the same association in two childhood age groups. Children (*N* = 280, age 3.5 years, *N* = 283, age 5 years) participated as part of the Longitudinal Israel Study of Twins. Affective knowledge was assessed through children’s responses to an illustrated story describing different emotional situations, told in a laboratory setting. The findings suggest a significant interaction between sex and the *DRD4-III* polymorphism, replicated in both age groups. Boy carriers of the 7R allele had higher affective knowledge scores than girls, whereas in the absence of the 7R there was no significant sex effect on affective knowledge. The results support the importance of *DRD4-III* polymorphism and sex differences to social development. Possible explanations for differences from adult findings are discussed, as are pathways for future studies.

## Introduction

*Affective knowledge*, the ability to understand others’ emotional states (e.g., Knafo et al., 2009), is important for children’s social functioning, and for the ability to communicate, cooperate, and cope with complex social interactions (Denham, 1986; Bauminger, 2002; Walker, 2005; Knafo et al., 2011b; Garner and Waajid, 2012).

Affective knowledge has been linked to *Empathy* – the tendency to share and understand the thoughts and feelings of others (Eisenberg and Strayer, 1990; Walter, 2012). Indeed, affective knowledge is often seen as an aspect of *Cognitive empathy*, the ability to recognize and understand what the other feels (Shamay-Tsoory et al., 2009). Cognitive empathy can also be defined in the framework of theory of mind (ToM) as, *Affective ToM*, the ability to represent and understand the affective mental states of others (Walter, 2012).

Affective knowledge, affective ToM, and cognitive empathy are all part of a network of interpersonal abilities that also includes *Affective empathy*, i.e., the ability to experience the emotion of the other while maintaining an emotional distinction between the self and the other (Decety and Lamm, 2006; de Vignemont and Singer, 2006). Studies in neuro-psychology and developmental psychology show that the two components are different, but not independent, aspects of the tendency to empathize (Singer, 2006; Volbrecht et al., 2007; Knafo et al., 2008a). In our literature review, therefore, we draw on evidence from the field of empathy research to understand the development of cognitive empathy and specifically affective knowledge.

Denham (1986) operationalized affective knowledge as composed of affective labeling (matching facial expressions to emotions) and affective perspective taking (matching a facial expression to someone based on their supposed emotional state). Garner and Waajid (2012) described affective knowledge as including awareness of emotion and knowledge of basic facial expressions (i.e., expression knowledge) and the situations that elicit emotions (situational knowledge). Similarly, many operationalizations of cognitive empathy (Matsumoto et al., 2000; Baron-Cohen et al., 2001), measured the ability to recognize an emotion from a facial expression, or predict an emotional response from a specific context. Based on these studies, our operationalization of affective knowledge (Knafo et al., 2009) combines aspects of these related approaches, by measuring attribution of emotional states, attribution of affective expressions, and matching facial expressions to attributed states.

### Sex Differences in Empathy and in Affective Knowledge

Studies typically show significant sex differences in empathy, with women scoring higher than men (Hoffman, 1977; Baron-Cohen and Wheelwright, 2004). Disorders associated with deficits in empathy and particularly in ToM (e.g., autism, Asperger syndrome; Baron-Cohen et al., 1997) are more prevalent in men than in women (Baron-Cohen and Wheelwright, 2004). Disorders such as depression and anxiety, that tend to be more prevalent in women, are associated with higher empathy (Zahn-Waxler et al., 1991, 2008). However, the association between sex and empathy is unclear, and may be dependent on the method used to measure empathy (Eisenberg and Lennon, 1983). Sex differences favoring females usually emerge using questionnaire measures (e.g., Adams et al., 1979), while performance measures usually do not yield sex differences (Hoffman, 1977; Eisenberg and Lennon, 1983). With respect to ToM, most investigations of individual differences in ToM did not specifically examine the issue of sex differences. Two exceptions are the studies of Charman et al. (2002) and of Walker (2005). Charman et al. (2002) investigated sex differences in false belief development and found a slight advantage for girls on false-belief task performance, an advantage that was only apparent in younger but not older children from a sample of 3–5 year-olds. In the second study, Walker (2005) showed that preschool girls are more competent than boys in ToM tasks, and that these sex differences were associated with peer-related social competence. Findings from studies that specifically focused on affective knowledge present a less than consistent picture (Hoffman, 1977; Eisenberg and Lennon, 1983; Gross and Ballif, 1991), with some studies showing a female advantage in affective knowledge tasks (Zahn-Waxler et al., 1984; Casey, 1993), and other studies finding no such effect (Cutting and Dunn, 1999).

These studies suggest that there are yet unanswered questions regarding the role of sex in explaining individual differences in empathy and affective knowledge, and that these differences may be important for understanding children’s social functioning outcomes.

### Heritability

There is much individual variation in empathic ability and specifically in affective knowledge – from extreme deficits, as in autism (Yirmiya et al., 1992) to differences in empathy and affective knowledge within the normal range seen in adults (Davis, 1980; Lawrence et al., 2004), children (Bryant, 1982; Denham, 1986; Knafo-Noam et al., 2015), and infants (Knafo et al., 2008a).

We are aware of a single study on affective ToM, in which 10-year-old’s recognition of facial expressions showed substantial heritability (Lau et al., 2009). Additional studies on children (Zhu et al., 2010) and adult population (McKone and Palermo, 2010; Wilmer et al., 2010) have found significant heritability effects for face recognition. Although we are not aware of additional published studies concerning the heritability of affective knowledge among children, it is possible to learn about its development from research on empathy and ToM.

#### Empathy

To the best of our knowledge, up to the time of writing this paper, there have been only eight twin studies addressing the genetic and environmental influences on individual differences in empathy, and in all of these studies (except one study which had extremely high correlations for both MZ and DZ twins) a significant genetic influence on empathy was found to exist from early childhood onward (Knafo and Uzefovsky, 2013). Our meta-analysis (Knafo and Uzefovsky, 2013), found that heritability accounts for 35% of the variance in empathy (the influence of the shared environment was negligible, and the influence of the non-shared environment, which also includes measurement error, was estimated as explaining 63% of individual variability). Interestingly, when examined separately, cognitive and affective empathy were found to have different patterns of genetic and environmental effects. Heritability explained 30% of the variance in affective empathy and 26% of the variance in cognitive empathy. Shared environment, estimated at 17%, explained individual variability in cognitive empathy only (the rest of the variance of both empathy facets was explained by non-shared environment and error, Knafo and Uzefovsky, 2013).

#### ToM

Similarly, there has been scant research estimating the genetic and environmental contributions to ToM. The available research shows moderate genetic as well as significant environmental contributions to individual differences in ToM (Ronald et al., 2005, 2006). A small study of 3.5 year-old twins showed a strong genetic effect on cognitive aspects of ToM (Hughes and Cutting, 1999). A larger study of 5-year-olds showed a genetic contribution to cognitive ToM, which overlapped with the genetic effects on language abilities (Hughes et al., 2005).

Taken together, the above-cited studies suggest that investigating the genetic basis of affective knowledge is a worthwhile endeavor.

### Specific Genetic Effects

Studies have shown that the social hormones oxytocin (OT) and vasopressin (AVP) facilitate and promote social interactions by modulating dopaminergic activity in the brain reward system (Young and Wang, 2004; Skuse and Gallagher, 2009). Relatedly, many molecular genetic studies have so far focused on the oxytocin receptor *(OXTR)* gene, repeatedly showing that variations in the *OXTR* are significantly associated with measures of empathy (Chakrabarti et al., 2009; Rodrigues et al., 2009; Wu et al., 2012; Lucht et al., 2013; Uzefovsky et al., 2015), as well as with difficulties in empathy (Schneiderman et al., 2013) and with autism (Chakrabarti et al., 2009) and ToM (Wu and Su, 2014). For a recent review, see Israel et al. (2015).

An additional recent study has also suggested that the association between genetic variation in *OXTR* and prosocial behavior is mediated by perspective taking and empathic concern, and that this pathway is contingent on sex (Christ et al., 2015). This study has shown an interaction between *OXTR* polymorphisms and sex in predicting prosocial tendencies (empathic concern and perspective taking), which in turn predict prosocial behavior. The patterns of genotype effects on prosocial tendencies were different for males and females (Christ et al., 2015). For example, the interaction between sex and rs2254298 showed that males with at least one A allele have significantly lower perspective taking scores compared to males who are homozygous for G allele, while no significant genotype effect in this polymorphism was found for females.

As noted, OT and AVP, together with activity of dopaminergic receptors, comprise an integrated neuronal system of social cognition (Skuse and Gallagher, 2009). The dopaminergic system is a critical component of the “social brain” which comprises areas of the brain that are involved in social cognition and behavior (Skuse and Gallagher, 2009). Studies have suggested that dopamine is crucial to empathy-motivated prosocial behavior, which has evolutionary roots in offspring care (Preston, 2013). Thus, the dopaminergic system is another good candidate system for investigating the genetic underpinnings of empathy. Since most studies to date have focused on the role of *OXTR* in empathy, there is still a big gap in the understanding of the role of dopamine, even though it is a crucial part of the social brain. Indeed, one recent study conducted on Chinese college students has shown that genetic variation in the dopamine system made significant contributions to individual differences in facial expression recognition, and specifically in the recognition of disgust faces (Zhu et al., 2011).

### DRD4 as a Candidate Gene for Affective Knowledge

*DRD4* is a gene that encodes the D4 receptor of dopamine and is one of the most studied candidate genes in relation to social behavior (Kang et al., 2008; Zhong et al., 2010). The gene has a number of variations (polymorphisms). One of the most researched polymorphisms of the gene is found in exon 3, which is characterized by a repeat region of 48 bp (translated to 16 amino acids) that can be repeated 2 – 11 times. This polymorphism (*DRD4-III*) was associated with behaviors and traits that are related to empathy. An example of this is the association between *DRD4-III* polymorphism and altruistic behavior (Bachner-Melman et al., 2005), and the possible role of the polymorphism in ADHD (Faraone et al., 2001), a disorder that was shown to be related to ToM deficits and reduced empathy (Uekermann et al., 2010). Another interesting investigation focused on the function of *DRD4-III* polymorphism in representational ToM (RTM) – the ability to explicitly understand that other’s mental states (beliefs, desires, knowledge) are person-specific representations of the world (Lackner et al., 2012). This study has suggested that variations in the *DRD4-III* may predict preschoolers’ performance in RTM, showing that individuals with two shorter alleles (4 repeats or less) outperformed those with one or two longer alleles (6 repeats or more), (Lackner et al., 2010).

In addition to these investigations, the *DRD4-III* has recently become the focus of research into gene by environment interactions (GxE). Carriers of the 7 repeat allele (7R), the second most common repeat in Caucasian populations (Chang et al., 1996) are thought to be more sensitive to environmental influence (Belsky and Pluess, 2009). An example of this is the study by Knafo et al. (2011a), which focused, using a sample that partially overlaps with the current sample, on prosocial behavior. Prosocial behavior is relevant in this context because many (but not all) prosocial behaviors are associated with empathy. Positive parenting was positively related to prosocial behavior (as reported by the mother), and unexplained punishment related positively to experimentally-elicited self-initiated pro-social behavior, but only for children carriers of the 7R allele. Carriers of other alleles showed no association between parenting and behavior (Knafo et al., 2011a). In a subsample of that study, mother-reported negativity toward the child was negatively associated with observed empathic concern toward an examiner, again only among children carriers of the 7R allele (Knafo and Uzefovsky, 2013).

One recent study examined the association between *DRD4-III* polymorphism and empathy among adults (Uzefovsky et al., 2014). A significant gene by sex interaction was found for cognitive empathy (but not emotional empathy), and was replicated in a second independent sample of adults. Specifically, it was found that women carriers of the 7R allele scored higher on cognitive empathy than women who were not carriers of the 7R allele, whereas for men, 7R carriers scored lower than non-carriers. This finding suggests that the polymorphism of *DRD4-III* is related to cognitive empathy and that sex differences are involved in this relationship.

### The Current Research

As cognitive empathy has been shown to have a genetic basis in early childhood (Knafo et al., 2009), one could expect to replicate the findings by Uzefovsky et al. (2014) among children. However, it is important to note the differences in measure type (cognitive empathy questionnaire vs. a task measuring affective knowledge) as well as the notion that genetic effects are often age-specific (e.g., Choh et al., 2014). For example, an AVPR1a polymorphism is associated with generosity in both adults and children, but different alleles are responsible for this association in the two age groups (Knafo et al., 2008b; Avinun et al., 2011). It is therefore important to study the role of genes in children, to get a developmental perspective on the association between the *DRD4-III* genotype, cognitive empathy, and specifically affective knowledge.

The current study sought to expand the knowledge of the genetic basis of the cognitive component of empathy to the developmental context. Specifically, we examined the association between the *DRD4-III* polymorphism and affective knowledge in children 3.5 and 5 years of age. In view of previous findings, this study focused on the *DRD4-III* 7R allele, hypothesizing that the association between genotype and affective knowledge would be contingent on sex. We expected to replicate the findings in adults (Uzefovsky et al., 2014) whereby sex interacted with the *DRD4-III* polymorphism in the association with cognitive empathy. However, as we are looking at a different age group and a different phenotype of social cognition we did not make strong hypotheses, but rather were interested in investigating the associations between *DRD4*-*III*, sex, and affective knowledge in early childhood.

## Materials and Methods

### Participants

Families in this study were participants in the Longitudinal Study of Twins (LIST) that focuses on children’s social development as influenced by genetics, abilities, and socialization (Knafo, 2006). All Hebrew-speaking families who were identified by the Israeli Ministry of the Interior as having twins born in 2004 and 2005, were contacted with mail surveys regarding children’s development close to the twins’ third birthday. See Avinun and Knafo (2013) for further details on the sample.

Families from the Greater Jerusalem area were invited to partake in an experimental session at the laboratory when twins reached 3.5 and 5 years of age. The laboratory session focused on evaluating empathy, pro-social behavior, cognitive abilities, and other social skills. In addition, DNA samples were taken from the twins and their parents, when parents’ agreement was obtained. The project was approved by the S. Herzog Hospital Institutional Review Board committee.

Of the initial lab sample we selected children with relevant data on both affective knowledge and DNA samples. Out of 447 individual participants from the first lab phase of the LIST, 128 were excluded because of a lack of DNA data, and 39 were excluded due to missing affective knowledge data. Similarly, out of 398 age 5 participants, 107 were excluded because of lack of DNA data, and eight were excluded due to missing affective knowledge data.

Therefore, the final sample from age 3.5 included 280 children (149 boys, 131 girls), aged 36–51 months (*M* = 44.13, SD = 2.78). The final age 5 sample included 283 children (149 boys, 134 girls), aged 59–71 months (*M* = 61.73, SD = 2.15). In total, 402 children participated at least once, of which 161 children participated in both phases.

### Procedure

Around the age of 3.5, families (the twins and a parent, sometimes accompanied by another family member) arrived at the lab for an experimental session. In the lab they met two examiners, and each twin was asked to enter a separate testing room with one of the examiners. Visits were scheduled at a time when parents estimated children were likely to be at their best. Assessments of social and cognitive development skills were made through a number of tasks (Avinun and Knafo, 2013), separately for each twin. Most visits lasted for less than 2 h. During or prior to the visit, mothers filled out questionnaires which included questions on the pregnancy, twins’ zygosity, twins’ behavior, and demographic details, including socioeconomic status.

Toward children’s fifth birthday all families from the twin sample living in the Greater Jerusalem area were invited to the lab again. The same laboratory procedure was performed when the twins were about 5 years old.

### Measures

*Affective knowledge* was measured using the Jerusalem Story Test of Interpersonal Understanding (Knafo et al., 2011b). This instrument consists of an illustrated story that taps several socio-cognitive abilities with a single story narrative loaded with various emotional associations (Knafo et al., 2011b). We followed Denham’s (1986, p. 194) recommendation, that to measure children’s affective knowledge a measure has to be sensitive to the needs of “capturing young children’s attention and of embedding tasks within an ongoing social context.” We therefore measured affective knowledge with children’s reactions to easy to understand situations, in a contextually valid setting of telling a story.

Stimuli and methods from existing relevant assessments (Denham, 1986; Ribordy et al., 1988) were integrated into the task. While reading the story, the experimenter asks the child predetermined questions tapping broad aspects of interpersonal understanding: affective knowledge, desire understanding, and false belief. Previously, measures from the test predicted children’s observed prosocial behavior (Knafo et al., 2011b) and empathy (Knafo et al., 2009). In the current investigation we focus on affective knowledge, as measured by the illustrated story assignment through three indices: emotion understanding, expression selection, and affective matching (see description below).

**FIGURE 1:**
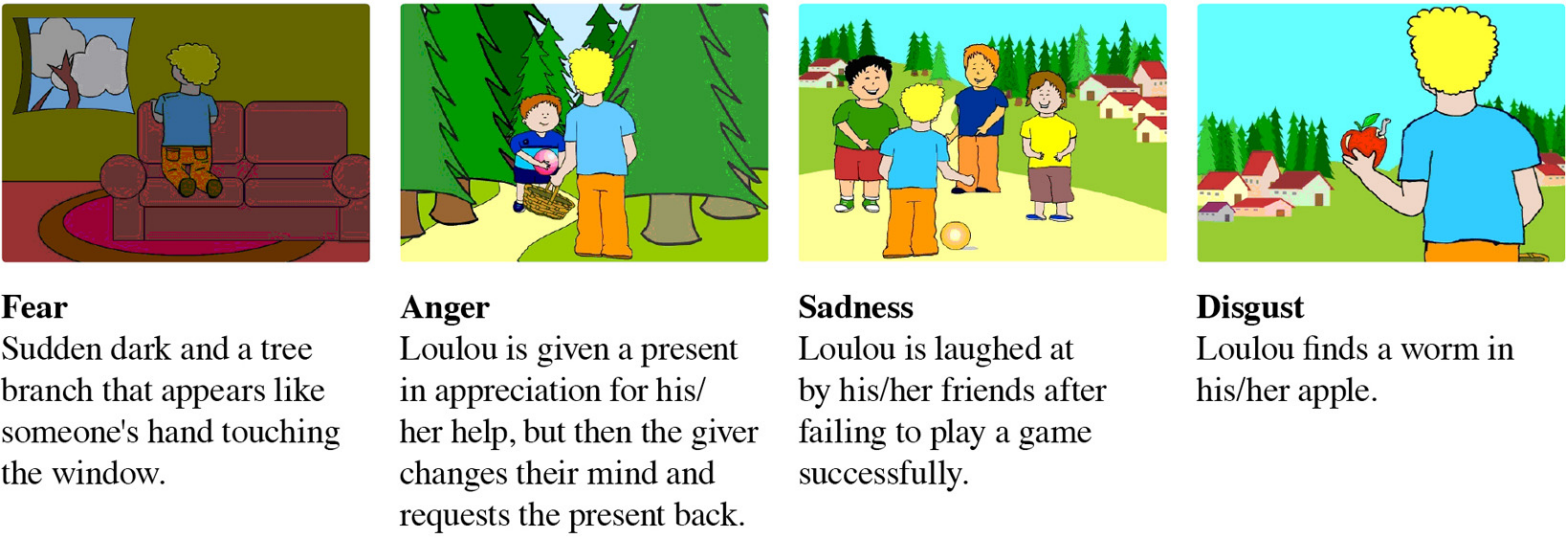
**An illustration of the four emotional situations described in the story**.

The story depicts emotional situations relevant to children’s lives, involving story character Loulou (matched to the participating child’s gender). Five situations (Ribordy et al., 1988) eliciting different emotions were examined: **happiness** (Loulou gets a long wished-for present), **fear** (a sudden darkness and a tree branch that appears like someone’s hand touching the window), **anger** (Loulou is given a present in appreciation for his/her help, but then the giver changes his mind and requests the present back), **sadness** (Loulou is laughed-at by his/her friends after failing to play a game successfully), and **disgust** (Loulou finds a worm in his/her apple), (see **Figure 1**). The four negative emotions were used in the current investigations following up on Knafo et al. (2009), in consideration of the differences between perceiving negative emotions of the other (Roberts and Strayer, 1996; Simpson et al., 2003; Eisenberg et al., 2014) and perceiving other’s positive emotions.

**FIGURE 2:**
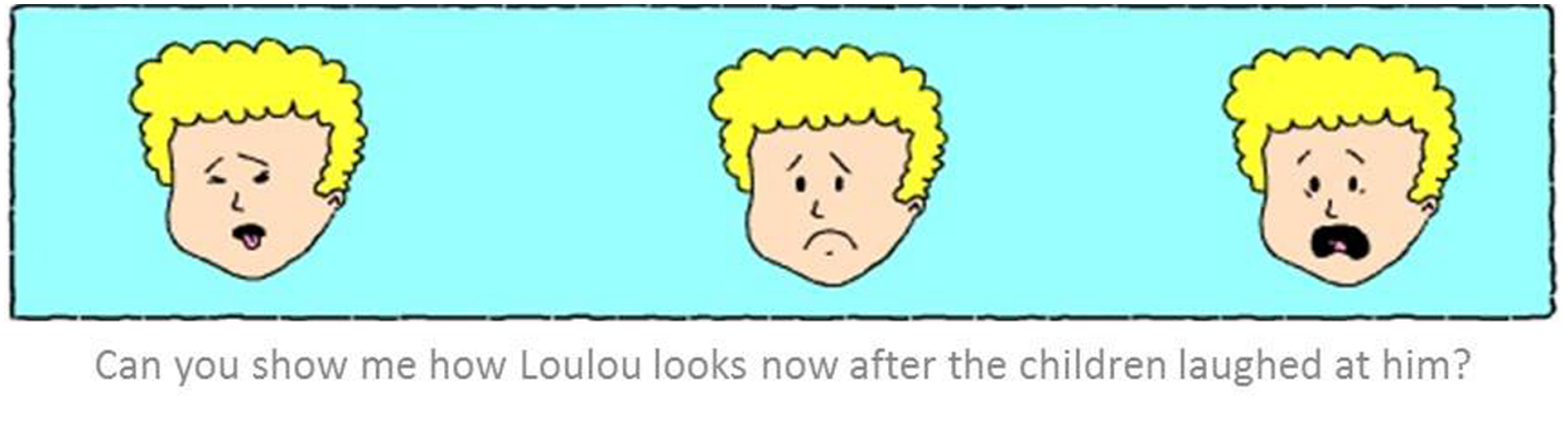
**Expression selection – an exemplary item assessing attribution of facial affective expression**.

Three measures of affective knowledge were obtained:

(1) *Emotion understanding – Attribution of emotional states:* After hearing each emotionally relevant situation, the child was asked, “How does Loulou feel?” If the child did not respond correctly or failed to respond at all, the question was repeated, and three options (e.g., angry, sad, yuck/disgusted) were presented. Responses were scored on a five-point scale: 4 = correct unaided answer; 3 = initial response concerning an overall negative emotion, which was correctly identified when options were given; 2 = wrong answer on unaided question and a correct answer when options were given; 1 = general negative affective response on unaided question and wrong answer when options were given; and 0 = wrong answer on both questions.
  The emotion understanding measure was calculated for each participant as a sum across the four situations, resulting in a score that could range between 0 and 16.
(2) *Expression selection – Attribution of facial affective expressions:* Following the previous question the child was shown three facial expressions of Loulou (corresponding to the three options given in the earlier question). For example, “can you show me how Loulou looks now after the children laughed at him/her?” (see **Figure 2**). A correct (matching the situation) answer was rated 3. Incorrect responses received a score between 2 and 0, depending on the degree of dissimilarity; previous research has shown that emotional expressions can be ordered in a circular manner based on the similarity in their different facial configurations (anger, disgust, happiness, surprise, fear, sadness, anger; Susskind et al., 2007). Incorrect answers were therefore scored according to the degree of difference between the face chosen and the correct face on this circumplex (e.g., anger and disgust involve two relatively similar facial configurations and confusing them would incur the score 2; in contrast, confusing the very different expressions of fear and disgust would incur the score 0).
  The expression selection measure was calculated for each participant as a sum across the four situations, resulting in a score that could range from 0 to 12.

(3) *Affective matching – Matching facial expressions to attributed states:* Success in matching label to facial expression was measured by counting the times in which the child chose a facial expression matching the emotion he or she named in response to the verbal question (“How does Loulou feel?”), regardless of whether this choice was correct or not with regards to the situation. Correct match was coded as 1, and an incorrect match as 0. A sum score was calculated, and could range between 0 and 4.

**Table 1** shows descriptive statistics for the three measures of affective knowledge. Each of the three indices of affective knowledge measures a different aspect of cognitive empathy. For example, a child could label an emotion correctly but fail to point to the right facial expression. In order to examine the factorial structure of all three measures we ran a factor analysis. As expected, since all three measures were designed to assess different aspects of affective knowledge, they were all significantly inter-correlated (*p* < 0.01, see correlations in **Table 2**) and loaded on a single factor. In the 3.5 year-olds sample, the factor accounted for 52.60% of the variance, with loadings ranging from 0.70 to 0.75. In the 5 year-olds sample the factor accounted for 52.43% of variance, with loadings ranging from 0.69 to 0.79. Thus, the factor structure remained relatively constant at both ages. Based on these results a total affective knowledge measure was computed by calculating *Z*-standardized scores for each measure, and averaging them. The descriptive statistics for the affective knowledge composite are shown in **Table 1**.

**Table 1:** Descriptive statistics for the affective knowledge composite and measures.

		*N*	Min	Max	*M*	SD	SE
3.5 years old	Emotion understanding	280	0	16	5.73	3.31	0.19
	Expression selection	280	2	12	7.73	2.07	0.12
	Affective matching	280	0	4	2.02	1.35	0.08
	**Affective knowledge**	**280**	**-1.46**	**1.9**	**0.01**	**0.69**	**0.04**
5 years old	Emotion understanding	283	0	16	8.40	3.04	0.18
	Expression selection	283	4	12	9.32	1.97	0.12
	Affective matching	283	0	4	2.69	1.27	0.07
	**Affective knowledge**	**283**	**-1.81**	**1.45**	**0.00**	**0.70**	**0.03**

The three measures comprising the affective knowledge score and the affective knowledge composite computed by standardizing the raw scores of the measures and averaging them.

**Table 2:** Pearson’s correlations.

		Expression selection	Affective matching
Emotion understanding	3.5 years old 5 years old	0.26^∗∗^ 0.29^∗∗^	0.27^∗∗^ 0.20^∗∗^
Expression selection	3.5 years old 5 years old		0.31^∗∗^ 0.33^∗∗^

^∗∗^*P* < 0.01.

#### *DRD4-III* Polymorphism

DNA was extracted from 20 ml of mouthwash samples using Master Pure kit (Epicentre, Madison, WI, USA). PCR (Polymerase chain reaction) amplification was carried. The exon III repeat region of the *DRD4* receptor was characterized using PCR amplification procedure (using a Reddy Mix kit, AB gene, Surrey, UK), and genotyping was conducted as previously described by Knafo et al. (2011a).

In the 3.5 years old sample 94 participants (33.57%) were carriers of the 7R allele, and 186 were non-carriers. In the 5 years old sample 79 participants (27.92%) were carriers of the 7R allele, and 204 were non-carriers. In both ages genotypes were in Hardy–Weinberg equilibrium as tested with the PEDSTATS software (Wigginton and Abecasis, 2005) for a sample in which one participant from each family was chosen randomly (Age 3.5: χ^2^ = 1.02, *p* = 0.79; Age 5: χ^2^ = 6.46, *p* = 0.09). Results reflect a stable frequency of the *DRD4* repeat alleles in the population under study (see **Table 3** for descriptive data regarding the distribution of the genotype by sex, for both ages).

**Table 3:** Frequencies of Genotype (7R+, 7R-) by sex (Boys, Girls).

		7R+	7R-	Total
3.5 Years old	Boys Girls	43 51	106 80	149 131
	**Total**	**94 (33.57%)**	**186 (66.43%)**	**280 (100%)**
5 years old	Boys Girls	38 41	111 93	149 134
	**Total**	**79 (27.92%)**	**204 (72.08%)**	**283 (100%)**

7R+: carries of the 7 repeat (7R) allele (Participants with the presence of at least one 7R allele).

7R-: non-carries of the 7R allele (Participants with the 7R allele absent).

### Statistical Analysis

The *DRD4-III* genotype was coded as a two level variable: “carriers of the 7R allele” (presence of at least one 7R allele), and “non-carriers of the 7R allele” (absence of a 7R allele), following Uzefovsky et al. (2014). Grouping of participants according to 7R-carrier status is common practice (e.g., Faraone et al., 2001; Bakermans-Kranenburg and van IJzendoorn, 2007; Knafo et al., 2011a; Uzefovsky et al., 2014) as the 7R allele is the second most common allele in Caucasian populations, and because of the difference in functionality between the 7R, specifically, and other alleles (Asghari et al., 1995). It would be interesting to test for an additive effect of sharing two 7R alleles, however, the homozygous (7-7R) genotype is relatively rare (in the current study 9 and 14 participants at ages 3.5 and 5, respectively).

The genotype, sex and their interaction served together as predictors of the affective knowledge score. Descriptive and preliminary statistics were carried out using SPSS v20 (Statistical Package for the Social Sciences). The main analyses were carried out using the GEE test (Generalized Estimating Equations test) in the SPSS. This test takes into account the dependency between twins, and enables using data from both twins. The results were verified by performing the analyses in the Mplus v5 software (Muthén and Muthén, 1998–2010). The procedure uses a slightly different analysis and a different set of assumptions to the GEE. Twins were considered as clustered within twin pairs, SE were computed using the TYPE = COMPLEX option, taking into account the fact that twin-data are non-independent of each other.

## Results

### Preliminary Analyses

Preliminary analyses showed that the sample of children, who participated at age 3.5 but not at age 5, did not differ on *DRD4* 7R genotype distribution, sex composition, or affective knowledge scores from those who participated at both time points. Similarly, children from families who joined the study at age 5 did not significantly differ from families who joined at age 3.5 on any study variable.

### DRD4-III 7R Polymorphism and Sex

The main hypothesis in our investigation was that the *DRD4-III* polymorphism would be associated with children’s affective knowledge, in a sex-contingent manner. We examined the association between the phenotype and the gene using the GEE procedure in SPSS v20. This procedure treats individual children as clustered by family using robust estimates of the SE, with affective knowledge regressed onto sex, *DRD4* genotype, and their interaction.

In the 3.5 years old sample the analysis yielded a significant effect of sex [Wald χ^2^(1, *N* = 280) = 4.53, *p* = 0.03], with boys scoring higher (*M* = 0.10, SE = 0.07) than girls (*M* = -0.09, SE = 0.06). Although there was no main effect for the *DRD4-III* polymorphism [Wald χ^2^(1, *N* = 280) = 0.605, *p* = 0.44], *DRD4-III* did qualify the sex difference, showing a significant interaction with sex [Wald χ^2^(1, *N* = 280) = 4.97, *p* = 0.02]. Results remained robust when the analysis was performed in the Mplus statistical package (β = -0.15, SE = 0.06, *p* = 0.013).

**FIGURE 3:**
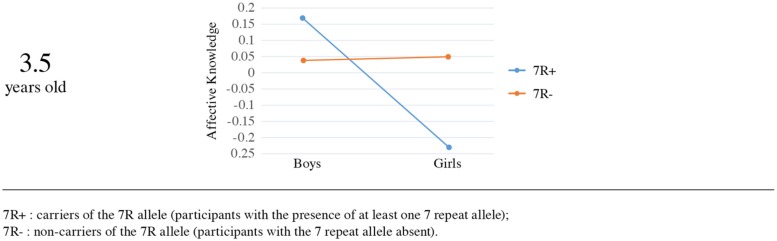
**The effect of *DRD4-III* polymorphism and sex on affective knowledge (3.5 years old)**.

In order to better understand the nature of the interaction we examined simple effects. Mean comparison indicated that among carriers of the 7R allele, boys had higher affective knowledge scores (*M* = 0.17, SE = 0.13) than girls (*M* = -0.23, SE = 0.07), whereas no such effect was found for non-carriers (*M* = 0.04, SE = 0.07 vs. *M* = 0.05, SE = 0.09, respectively; see **Figure 3**). The sex effect on affective knowledge was significant for carriers of the 7R allele [Wald χ^2^(1, *N* = 280) = 7.66, *p* = 0.006], but not among non-carriers [Wald χ^2^(1, *N* = 280) = 0.01, *p* = 0.92]. Examination of the genotype effect separately for boys and girls yielded a significant effect among girls, as the 7R allele was significantly associated with lower affective knowledge scores and the absence of the 7R was significantly associated with higher affective knowledge scores [Wald χ^2^(1, *N* = 280) = 5.85, *p* = 0.016]. No significant genotype effect was found for boys [Wald χ^2^(1, *N* = 280) = 0.88, *p* = 0.35].

In an attempt to check whether these results are consistent in two different ages through childhood, we examined the association between the phenotype and the gene in the 5 year-olds sample. The main effects of genotype [Wald χ^2^(1, *N* = 283) = 1.08, *p* = 0.30] and sex [Wald χ^2^(1, *N* = 283) = 0.28, *p* = 0.59] were not significant, but the interaction between sex and *DRD4-III* polymorphism was again significant [Wald χ^2^(1, *N* = 283) = 4.238, *p* = 0.04], suggesting that the effect of the gene on affective knowledge is moderated by sex in both age groups. The interaction was significant in the Mplus analysis as well (β = -0.12, SE = 0.06, *p* = 0.039).

**FIGURE 4:**
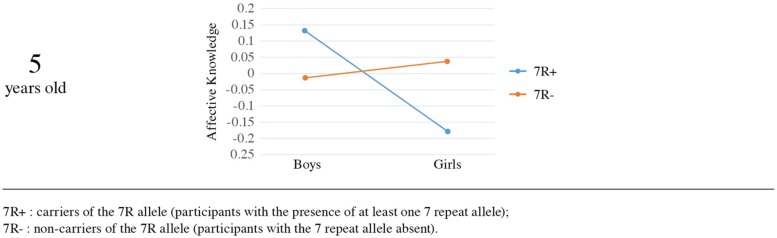
**The effect of *DRD4-III* polymorphism and sex on affective knowledge (5 years old)**.

Furthermore, the direction of effects was similar in both ages. We examined simple effects in order to understand the nature of the interaction. Mean comparison indicated that among carriers of the 7R allele, boys had higher affective knowledge scores (*M* = 0.13, SE = 0.11) than girls (*M* = -0.18, SE = 0.09), whereas no such effect was found for non-carriers (*M* = -0.01, SE = 0.08 vs. *M* = 0.04, SE = 0.08, respectively; see **Figure 4**). The sex effect on affective knowledge was significant for carriers of the 7R allele [Wald χ^2^(1, *N* = 283) = 4.816, *p* = 0.03], showing that in the presence of the 7R allele boys scored significantly higher (*M* = 0.13, SE = 0.11) than girls (*M* = -0.18, SE = 0.09). For non-carriers of the 7R there was no significant sex effect on affective knowledge [Wald χ^2^(1, *N* = 283) = 0.28, *p* = 0.56]. Examination of the genotype effect separately for boys and girls yielded no significant effect for boys [Wald χ^2^(1, *N* = 283) = 1.08, *p* = 0.30] or for girls [Wald χ^2^(1, *N* = 283) = 3.23, *p* = 0.07].

**FIGURE 5:**
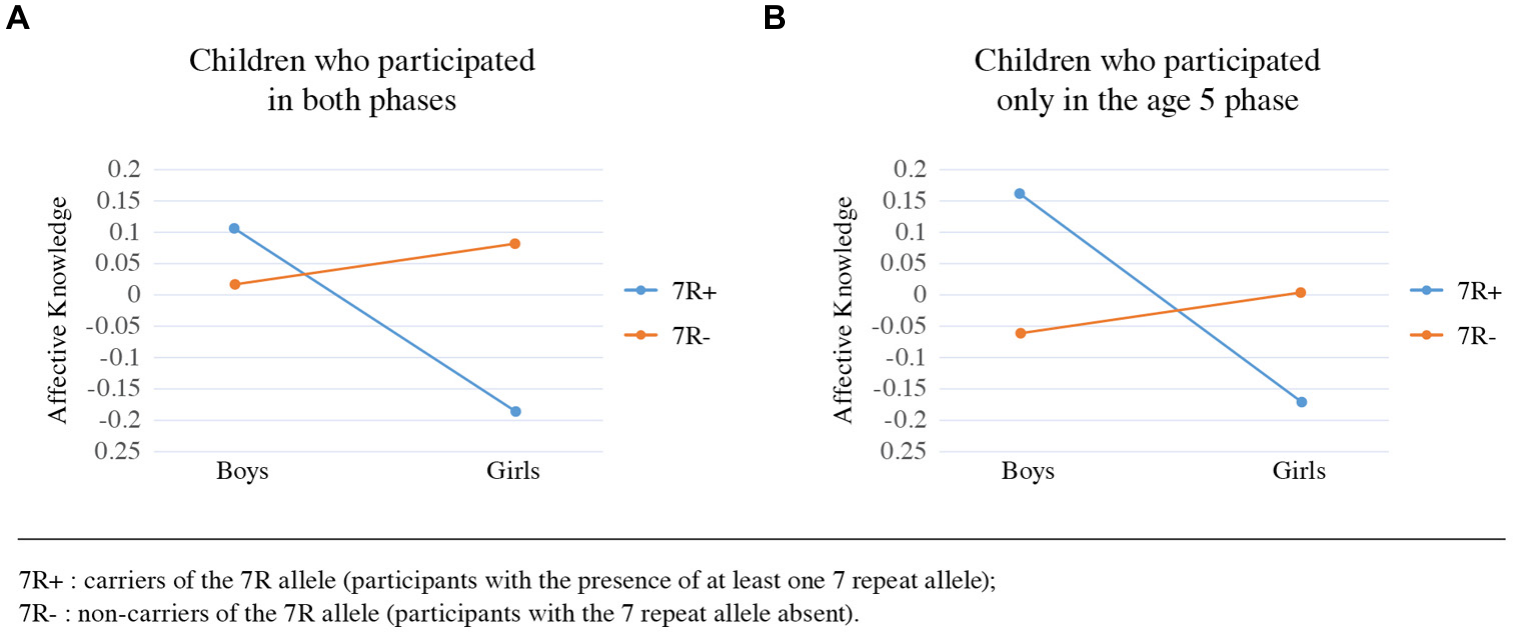
**Affective knowledge by *DRD4-III* polymorphism and sex of subsample of children.** Mean comparison showing the same pattern of findings for children who participated in the 3.5 years old phase and retained for the 5 years old phase **(A)** and for children who participated only in the 5 years old phase **(B)**.

In a subsample of children who only participated in the age 5 phase there was no significant effect for either sex, *DRD4-III* or their interaction, possibly reflecting the small sample size for this group (*N* = 121). Nevertheless, it is important to note that the pattern of findings, although not significant, was very similar for children from this new sample and for those retained from the age 3 phase, as seen in **Figure 5**.

Because there were age differences within each age group, and to account for the possible role of children’s social and developmental background, we further tested several potential contributing variables. Gestational age (in weeks) or birthweight did not relate significantly to affective knowledge at either age group. Within-age group age differences (in months) were associated with affective knowledge at age 3.5 (*r* = 0.16, *p* = 0.01), and more weakly so at 5 (*r* = 0.10, *ns*). Family socio-economic status (SES, indicated by mothers’ report on the family’s income relative to the given national average, where 1 = much below national average, 3 = around average, and 5 = much above average) did not significantly correlate with affective knowledge at age 3.5 (*r* = -0.12, *ns*), but was significantly related to better performance at age 5 (*r* = 0.25, *p* = 0.01).

We therefore examined a covariate-adjusted model, controlling for within-age group age differences and SES. Importantly, the genotype × sex interaction remained significant when controlling for these variables, at both age 3.5, [Wald χ^2^(1, *N* = 236) = 5.00 *p* = 0.025], and age 5 [Wald χ^2^(1, *N* = 210) = 3.92, *p* = 0.048], attesting to the robustness of the findings.

## Discussion

We examined the association between the *DRD4-III* polymorphism and affective knowledge among children 3.5 and 5 years of age. The findings demonstrate that the association between *DRD4-III* and affective knowledge is contingent on sex. In both age groups, in the presence of the 7R allele boys scored significantly higher than girls, whereas in the absence of the 7R there was no significant sex effect on affective knowledge.

Due to the fact that socio-cognitive abilities, and especially cognitive empathy, develop dramatically during the preschool period (Lennon and Eisenberg, 1990), this consistent replication across two age groups is of special value. In other words, although children mature and change in these critical years, the interaction effect remains consistent, reflecting its robustness.

The results of the current study can be interpreted in the context of brain mechanisms underlying socio-cognitive abilities. The *DRD4* is widely expressed in the brain, particularly in the prefrontal cortex, hippocampus, hypothalamus, amygdala, and mesolimbic pathways (Matsumoto et al., 1995; Oak et al., 2000). These regions are considered to be a part of the “social brain” (Skuse and Gallagher, 2009), that includes the amygdala as one of the central parts of the reward system. *DRD4* is an integral part of the dopamine system, a system that is considered to be involved in making social interactions rewarding. The importance of the dopaminergic reward circuits in the regulation of social cognition is presented in the model of Skuse and Gallagher (2009, 2011). This model suggests that genetic variation in the receptors associated with OT, AVP, and dopamine may explain individual differences, as well as, deficits, in socio-cognitive processes and behaviors (Skuse and Gallagher, 2009, 2011). For example, the negative symptoms of schizophrenia (impairments in emotional processing, social perception and knowledge, ToM, and attributional bias) may be associated with abnormalities in OT and dopamine signaling in the amygdala (Rosenfeld et al., 2011). In this context it is important to note that the *DRD4* specifically was found to be expressed in excess in the striatum in postmortem brains of schizophrenia patients (Seeman et al., 1993). The current findings add to the literature by showing that dopamine, and especially *DRD4*, have an important role in typical social cognition, as well as in psychopathology.

Interestingly, the direction of the sex by gene interaction effect on affective knowledge was reversed compared to the effect found in adult populations on cognitive empathy (Uzefovsky et al., 2014). What may be the reasons for this reversal? One possibility is that genetic effects may be different across different developmental stages. Genetic effects can change from childhood to adulthood, both in the overall heritability of a trait (Haworth et al., 2008, 2010), and in the specific molecular genetic contributions to individual variability, as discussed above with regards to the genetics of generosity (Knafo et al., 2008b; Avinun et al., 2011). In addition, we must consider the fact that the sex effect can reflect both biological sex and/or gender, the social aspect of sex. Therefore, the gene by sex interaction can be understood in different ways (as elaborated by Uzefovsky et al., 2014).

Considering sex effects as related to biological as well as to social mechanisms, it is reasonable to assume that the effects of these mechanisms change from childhood to adulthood. Significant biological and social changes occur in the gap between these age periods, especially during adolescence. For example, the body undergoes significant externally visible changes due to surges in sex-hormones during puberty, and adolescents experience an increase in gender-differential socialization pressure (Hill and Lynch, 1983). Studies have shown that social behavior, such as pair bonding, romantic relationship, concern for others, and empathy, is influenced by sex-hormones (Hastings et al., 2006; van Anders and Gray, 2007). There are also some empirical data that support the notion that socialization contributes to gender differences in empathy (Lennon and Eisenberg, 1990). Taken together, it is possible that after a long period of gender-related changes (social and/or biological) the interactive contribution of gene and sex on cognitive empathy may change. These might explain the reversed direction of interaction between the different populations (children and adults).

Moreover, unless they are measured in the same sample, age differences often represent the fact that individuals of different ages were born and raised in different periods (i.e., cohort effects, e.g., Smits et al., 2011). The children in the present study were born two decades after the participants of the adult study (Uzefovsky et al., 2014), and changes in gender roles during this period may account in part for this difference (Twenge, 1997).

Finally, it is important to mention here that beyond the age difference between participants in the study of Uzefovsky et al. (2014) and the current study, there are also differences in the method used to assess the dependent variable. While adults’ empathy was measured using a self-report questionnaire, in the current study we used a performance-based measure of affective knowledge. Although self-reported measures can reveal a complex and stable characteristic, they are more sensitive to reporter bias and demand characteristics. This might have an additional influence on the different findings.

Raising the possible reasons for the reversal in the interaction direction still leaves us with many unsolved questions: What is it about being a child-boy that, in the presence of the 7R allele, predisposes to higher affective knowledge scores? Why and when does this predisposition change during the maturational processes? Addressing these questions is very important and challenging due to the elusive nature of the notion “sex,” as we note above. Carriers of the 7R allele are thought to be sensitive to environmental influence (e.g., Belsky and Pluess, 2009), and thus a combined effect of socialization effects across development with their tendency for being more strongly affected by such effects could contribute to the developmental change in the observed genotype × sex effects.

### Conclusions and Suggestions for Future Research

In line with previous studies showing that the dopaminergic system is essential for social cognition, our findings suggest that *DRD4-III* polymorphism is associated with affective knowledge starting from early childhood, in interaction with sex. Being the first study to investigate the association between *DRD4-III* polymorphism and affective knowledge among children, this study provides novel evidence for the particular association of the genotype with cognitive empathy in early childhood. A comparison with a recent study conducted on adult population reveals the possibility that the direction of the gender effect on the association between the genotype and phenotype changes throughout development.

Further studies are crucial in order to validate these findings and to expand our understanding of the molecular genetics of empathy and related variables. Future studies should use multiple measures, to better understand the role of measurement type on the results. In addition, it is important to examine the interaction effect of *DRD4-III* polymorphism and sex on the emotional component of empathy among children. Finally, the current findings emphasize the need to examine the role of genes in various age groups, from childhood, through adolescence to adulthood. This is especially important for understanding the reported interaction to determine whether and when the direction of the gender effect changes.

## Conflict of Interest Statement

The authors declare that the research was conducted in the absence of any commercial or financial relationships that could be construed as a potential conflict of interest.

## References

[B1] Adams G. R. Schvaneveldt J. D. Jenson G. O. (1979). Sex, age and perceived competency as correlates of empathic ability in adolescence. *Adolescence* 14 811–818.

[B2] Asghari V. Sanyal S. Buchwaldt S. Paterson A. Jovanovic V. Van Tol H. H. (1995). Modulation of intracellular cyclic AMP levels by different human dopamine D4 receptor variants. *J. Neurochem.* 65 1157–1165. 10.1046/j.1471-4159.1995.65031157.x 7643093

[B3] Avinun R. Israel S. Shalev I. Gritsenko I. Bornstein G. Ebstein R. P. (2011). AVPR1A variant associated with preschoolers’ lower altruistic behavior. *PLoS ONE* 6:e25274 10.1371/journal.pone.0025274 PMC3182215 21980412

[B4] Avinun R. Knafo A. (2013). The longitudinal Israeli study of twins (LIST) – an integrative view of social development. *Twin Res. Hum. Genet.* 16 197–201. 10.1017/thg.2012.73 23394191

[B5] Bachner-Melman R. Gritsenko I. Nemanov L. Zohar A. H. Dina C. Ebstein R. P. (2005). Dopaminergic polymorphisms associated with self-report measures of human altruism: a fresh phenotype for the dopamine D4 receptor. *Mol. Psychiatry* 10 333–335. 10.1038/sj.mp.4001635 15655563

[B6] Bakermans-Kranenburg M. J. van IJzendoorn M. H. (2007). Research review: genetic vulnerability or differential susceptibility in child development: the case of attachment. *J. Child Psychol. Psychiatry* 48 1160–1173. 10.1111/j.1469-7610.2007.01801.x 18093021

[B7] Baron-Cohen S. Jolliffe T. Mortimore C. Robertson M. (1997). Another advanced test of theory of mind: evidence from very high functioning adults with autism or Asperger syndrome. *J. Child Psychol. Psychiatry* 38 813–822. 10.1111/j.1469-7610.1997.tb01599.x 9363580

[B8] Baron-Cohen S. Wheelwright S. (2004). The empathy quotient: an investigation of adults with Asperger syndrome or high functioning autism, and normal sex differences. *J. Autism. Dev. Disord.* 34 163–175. 10.1023/B:JADD.0000022607.19833.00 15162935

[B9] Baron-Cohen S. Wheelwright S. Hill J. Raste Y. Plumb I. (2001). The “Reading the mind in the eyes” test revised version: a study with normal adults, and adults with Asperger syndrome or high-functioning autism. *J. Child Psychol. Psychiatry* 42 241–251. 10.1111/1469-7610.00715 11280420

[B10] Bauminger N. (2002). The facilitation of social-emotional understanding and social interaction in high-functioning children with autism: intervention outcomes. *J. Autism. Dev. Disord.* 32 283–298. 10.1023/A:1016378718278 12199133

[B11] Belsky J. Pluess M. (2009). Beyond diathesis stress: differential susceptibility to environmental influences. *Psychological. Bull.* 135 885–908. 10.1037/a0017376 19883141

[B12] Bryant B. K. (1982). An index of empathy for children and adolescents. *Child Dev.* 53 413–425. 10.2307/1128984

[B13] Casey R. J. (1993). Children’s emotional experience: relations among expression, self-report, and understanding. *Dev. Psychol.* 29 119–129. 10.1037/0012-1649.29.1.119

[B14] Chakrabarti B. Dudbridge F. Kent L. Wheelwright S. Hill-Cawthorne G. Allison C. (2009). Genes related to sex steroids, neural growth, and social-emotional behavior are associated with autistic traits, empathy, and asperger syndrome. *Autism Res.* 2 157–177. 10.1002/aur.80 19598235

[B15] Chang F. M. Kidd J. R. Livak K. J. Pakstis A. J. Kidd K. K. (1996). The world-wide distribution of allele frequencies at the human dopamine D4 receptor locus. *Hum. Genet.* 98 91–101. 10.1007/s004390050166 8682515

[B16] Charman T. Ruffman T. Clements W. (2002). Is there a gender difference in false belief development? *Soc. Dev.* 11 1–10. 10.1111/1467-9507.00183

[B17] Choh A. C. Lee M. Kent J. W. Diego V. P. Johnson W. Curran J. E. (2014). Gene-by-age effects on BMI from birth to adulthood: the fels longitudinal study. *Obesity* 22 875–881. 10.1002/oby.20517 23794238 PMC3883986

[B18] Christ C. C. Carlo G. Stoltenberg S. F. (2015). Oxytocin receptor (OXTR) single nucleotide polymorphisms indirectly predict prosocial behavior through perspective taking and empathic concern. *J. Pers.* 10.1111/jopy.12152 [Epub ahead of print]. 25403479

[B19] Cutting A. L. Dunn J. (1999). Theory of mind, emotion understanding, language, and family background: individual differences and interrelations. *Child Dev.* 70 853–865. 10.1111/1467-8624.00061 10446724

[B20] Davis M. (1980). A multidimensional approach to individual differences in empathy. *JSAS Catal. Select. Doc. Psychol.* 10 85–104.

[B21] Decety J. Lamm C. (2006). Human empathy through the lens of social neuroscience. *Sci. World J.* 6 1146–1163. 10.1100/tsw.2006.221 PMC5917291 16998603

[B22] Denham S. A. (1986). Social cognition, prosocial behavior, and emotion in preschoolers: contextual validation. *Child Dev.* 57 194–201. 10.2307/1130651

[B23] de Vignemont F. Singer T. (2006). The empathic brain: how, when and why? *Trends Cogn. Sci.* 10 435–441. 10.1016/j.tics.2006.08.008 16949331

[B24] Eisenberg N. Lennon R. (1983). Sex differences in empathy and related capacities. *Psychol. Bull.* 94 100–131. 10.1037/0033-2909.94.1.100

[B25] Eisenberg N. Shea C. L. Carlo G. Knight G. P. (2014). “Empathy-related responding and cognition: a chicken and the egg dilemma,” in *Handbook of Moral Behavior and Development Research* Vol. 2 eds Kurtines W. M. Gewirtz J. L. (New York: Erlbaum).

[B26] Eisenberg N. Strayer J. (1990). “Critical issues in the study of empathy,” in *Empathy and Its Development* eds Eisenberg N. Strayer J. (Cambridge: Cambridge University Press) 7–13.

[B27] Faraone S. V. Doyle A. E. Mick E. Biederman J. (2001). Meta-analysis of the association between the 7-repeat allele of the dopamine D4 receptor gene and attention deficit hyperactivity disorder. *Am. J. Psychiatry* 158 1052–1057. 10.1176/appi.ajp.158.7.1052 11431226

[B28] Garner P. W. Waajid B. (2012). Emotion knowledge and self-regulation as predictors of preschoolers’ cognitive ability, classroom behavior, and social competence. *J. Psychoeduc. Assess.* 30 330–343. 10.1177/0734282912449441

[B29] Gross A. L. Ballif B. (1991). Children’s understanding of emotion from facial expressions and situations: a review. *Dev. Rev.* 11 368–398. 10.1016/0273-2297(91)90019-K

[B30] Hastings P. D. Zahn-Waxler C. McShane K. (2006). “We are, by nature, moral creatures: biological bases of concern for others,” in *Handbook of Moral Development* ed. Smetana M. K. J. G. (Mahwah, NJ: Erlbaum Publisher) 483–516.

[B31] Haworth C. Carnell S. Meaburn E. L. Davis O. S. Plomin R. Wardle J. (2008). Increasing heritability of BMI and stronger associations with the FTO gene over childhood. *Obesity* 16 2663–2668. 10.1038/oby.2008.434 18846049

[B32] Haworth C. M. A. Wright M. J. Luciano M. Martin N. G. De Geus E. J. C. Van Beijsterveldt C. E. M. (2010). The heritability of general cognitive ability increases linearly from childhood to young adulthood. *Mol. Psychiatry* 15 1112–1120. 10.1038/mp.2009.55 19488046 PMC2889158

[B33] Hill J. P. Lynch M. E. (1983). “The intensification of gender-related role expectations during early adolescence,” in *Girls at Puberty* eds Brooks-Gunn J. (Berlin: Springer) 201–228.

[B34] Hoffman M. L. (1977). Sex differences in empathy and related behaviors. *Psychol. Bull.* 84 712–722. 10.1037/0033-2909.84.4.712 897032

[B35] Hughes C. Cutting A. L. (1999). Nature, nurture, and individual differences in early understanding of mind. *Psychol. Sci.* 10 429–432. 10.1111/1467-9280.00181

[B36] Hughes C. Jaffee S. R. Happé F. Taylor A. Caspi A. Moffitt T. E. (2005). Origins of individual differences in theory of mind: from nature to nurture? *Child Dev.* 76 356–370. 10.1111/j.1467-8624.2005.00850_a.x 15784087

[B37] Israel S. Hasenfratz L. Knafo-Noam A. (2015). The genetics of morality and prosociality. *Curr. Opin. Psychol.* 6 55–59. 10.1016/j.copsyc.2015.03.027

[B38] Kang J. I. Namkoong K. Kim S. J. (2008). Association of DRD4 and COMT polymorphisms with anger and forgiveness traits in healthy volunteers. *Neurosci. Lett.* 430 252–257. 10.1016/j.neulet.2007.11.005 18063308

[B39] Knafo A. (2006). The longitudinal Israeli study of twins (LIST): children’s social development as influenced by genetics, abilities, and socialization. *Twin Res. Hum. Genet.* 9 791–798. 10.1375/twin.9.6.791 17254410

[B40] Knafo A. Israel S. Ebstein R. P. (2011a). Heritability of children’s prosocial behavior and differential susceptibility to parenting by variation in the dopamine receptor D4 gene. *Dev. Psychopathol.* 23 53–67. 10.1017/S0954579410000647 21262039

[B41] Knafo A. Steinberg T. Goldner I. (2011b). Children’s low affective perspective-taking ability is associated with low self-initiated pro-sociality. *Emotion* 11 194–198. 10.1037/a0021240 21401240

[B42] Knafo A. Uzefovsky F. (2013). “Variation in empathy: the interplay of genetic and environmental factors,” in: *The Infant Mind: Origins of the Social Brain* eds Legerstee M. Haley D. W. Bornstein M. H. (New York: The Guilford Press) 97–122.

[B43] Knafo A. Zahn-Waxler C. Davidov M. Van Hulle C. Robinson J. L. Rhee S. H. (2009). Empathy in early childhood. *Ann. N. Y. Acad. Sci.* 1167 103–114. 10.1111/j.1749-6632.2009.04540.x 19580557

[B44] Knafo A. Zahn-Waxler C. Van Hulle C. Robinson J. L. Rhee S. H. (2008a). The developmental origins of a disposition toward empathy: genetic and environmental contributions. *Emotion* 8 737–752. 10.1037/a0014179 19102585

[B45] Knafo A. Israel S. Darvasi A. Bachner-Melman R. Uzefovsky F. Cohen L. (2008b). Individual differences in allocation of funds in the Dictator Game and postmortem hippocampal mRNA levels are correlated with length of the arginine vasopressin 1a receptor (AVPR1a) RS3 promoter-region repeat. *Genes Brain Behav.* 7 266–275. 10.1111/j.1601-183X.2007.00341.x 17696996

[B46] Knafo-Noam A. Uzefovsky F. Israel S. Davidov M. Zahn-Waxler C. (2015). The prosocial personality and its facets: genetic and environmental architecture of mother-reported behavior of 7-year-old twins. *Front Psychol.* 6:112 10.3389/fpsyg.2015.00112 PMC4327421 25762952

[B47] Lackner C. L. Bowman L. C. Sabbagh M. A. (2010). Dopaminergic functioning and preschoolers’ theory of mind. *Neuropsychologia* 48 1767–1774. 10.1016/j.neuropsychologia.2010.02.027 20206642

[B48] Lackner C. Sabbagh M. A. Hallinan E. Liu X. Holden J. J. (2012). Dopamine receptor D4 gene variation predicts preschoolers’ developing theory of mind. *Dev. Sci.* 15 272–280. 10.1111/j.1467-7687.2011.01124.x 22356182

[B49] Lau J. Y. Burt M. Leibenluft E. Pine D. S. Rijsdijk F. Shiffrin N. (2009). Individual differences in children’s facial expression recognition ability: the role of nature and nurture. *Dev. Neuropsychol.* 34 37–51. 10.1080/87565640802564424 19142765 PMC2797312

[B50] Lawrence E. J. Shaw P. Baker D. Baron-Cohen S. David A. S. (2004). Measuring empathy: reliability and validity of the Empathy Quotient. *Psychol. Med.* 34 911–920. 10.1017/S0033291703001624 15500311

[B51] Lennon R. Eisenberg N. (1990). “Gender and age differences in empathy and sympathy,” in *Empathy and its Development* eds Eisenberg N. Strayer J. (Cambridge: Cambridge University Press) 195–213.

[B52] Lucht M. J. Barnow S. Sonnenfeld C. Ulrich I. Grabe H. J. Schroeder W. (2013). Associations between the oxytocin receptor gene (OXTR) and “mind-reading” in humans – an exploratory study. *Nord. J. Psychiatry* 67 15–21. 10.3109/08039488.2012.700731 22809402

[B53] Matsumoto D. LeRoux J. Wilson-Cohn C. Raroque J. Kooken K. Ekman P. (2000). A new test to measure emotion recognition ability: matsumoto and Ekman’s Japanese and caucasian brief affect recognition test (JACBART). *J. Nonverbal Behav.* 24 179–209. 10.1023/A:1006668120583

[B54] Matsumoto M. Hidaka K. Tada S. Tasaki Y. Yamaguchi T. (1995). Full-length cDNA cloning and distribution of human dopamine D4 receptor. *Mol. Brain Res.* 29 157–162. 10.1016/0169-328X(94)00245-A 7769992

[B55] McKone E. Palermo R. (2010). A strong role for nature in face recognition. *Proc. Natl. Acad. Sci.* 107 4795–4796. 10.1073/pnas.1000567107 20212105 PMC2841894

[B56] Muthén L. K. Muthén B. O. (1998–2010). *Mplus User’s Guide* 6th edn Los Angeles, CA: Authors.

[B57] Oak J. N. Oldenhof J. Van Tol H. H. (2000). The dopamine D 4 receptor: one decade of research. *Eur. J. Pharmacol.* 405 303–327. 10.1016/S0014-2999(00)00562-8 11033337

[B58] Preston S. D. (2013). The origins of altruism in offspring care. *Psychol. Bull.* 139 1305–1341. 10.1037/a0031755 23458432

[B59] Ribordy S. C. Camras L. A. Stefani R. Spaccarelli S. (1988). Vignettes for emotion recognition research and affective therapy with children. *J. Clin. Child Psychol.* 17 322–325. 10.1207/s15374424jccp1704_4

[B60] Roberts W. Strayer J. (1996). Empathy, emotional expressiveness, and prosocial behavior. *Child Dev.* 67 449–470. 10.2307/1131826

[B61] Rodrigues S. M. Saslow L. R. Garcia N. John O. P. Keltner D. (2009). Oxytocin receptor genetic variation relates to empathy and stress reactivity in humans. *Proc. Natl. Acad. Sci. U.S.A.* 106 21437–21441. 10.1073/pnas.0909579106 19934046 PMC2795557

[B62] Ronald A. Happé F. Hughes C. Plomin R. (2005). Nice and nasty theory of mind in preschool children: nature and nurture. *Soc. Dev.* 14 664–684. 10.1111/j.1467-9507.2005.00323.x

[B63] Ronald A. Viding E. Happé F. Plomin R. (2006). Individual differences in theory of mind ability in middle childhood and links with verbal ability and autistic traits: a twin study. *Soc. Neurosci.* 1 412–425. 10.1080/17470910601068088 18633802

[B64] Rosenfeld A. J. Lieberman J. A. Jarskog L. F. (2011). Oxytocin, dopamine, and the amygdala: a neurofunctional model of social cognitive deficits in schizophrenia. *Schizophr. Bull.* 37 1077–1087. 10.1093/schbul/sbq015 20308198 PMC3160224

[B65] Schneiderman I. Kanat-Maymon Y. Ebstein R. P. Feldman R. (2013). Cumulative risk on the oxytocin receptor gene (OXTR) underpins empathic communication difficulties at the first stages of romantic love. *Soc. Cogn. Affect. Neurosci.* 1524–1529. 10.1093/scan/nst142 23974948 PMC4187267

[B66] Seeman P. Guan H. C. Van Tol H. H. (1993). Dopamine D4 receptors elevated in schizophrenia. *Nature* 365 441–445. 10.1038/365441a0 8413587

[B67] Shamay-Tsoory S. G. Aharon-Peretz J. Perry D. (2009). Two systems for empathy: a double dissociation between emotional and cognitive empathy in inferior frontal gyrus versus ventromedial prefrontal lesions. *Brain* 132 617–627. 10.1093/brain/awn279 18971202

[B68] Simpson J. A. Oriña M. M. Ickes W. (2003). When accuracy hurts, and when it helps: a test of the empathic accuracy model in marital interactions. *J. Pers. Soc. Psychol.* 85 881–893. 10.1037/0022-3514.85.5.881 14599251

[B69] Singer T. (2006). The neuronal basis and ontogeny of empathy and mind reading: review of literature and implications for future research. *Neurosci. Biobehav. Rev.* 30 855–863. 10.1016/j.neubiorev.2006.06.011 16904182

[B70] Skuse D. H. Gallagher L. (2009). Dopaminergic-neuropeptide interactions in the social brain. *Trends Cogn. Sci.* 13 27–35. 10.1016/j.tics.2008.09.007 19084465

[B71] Skuse D. H. Gallagher L. (2011). Genetic influences on social cognition. *Pediatr. Res.* 69 85–91. 10.1203/PDR.0b013e318212f562 21289535

[B72] Smits I. A. Dolan C. V. Vorst H. Wicherts J. M. Timmerman M. E. (2011). Cohort differences in Big Five personality factors over a period of 25 years. *J. Pers. Soc. Psychol.* 100 1124–1138. 10.1037/a0022874 21534699

[B73] Susskind J. M. Littlewort G. Bartlett M. S. Movellan J. Anderson A. K. (2007). Human and computer recognition of facial expressions of emotion. *Neuropsychologia* 45 152–162. 10.1016/j.neuropsychologia.2006.05.001 16765997

[B74] Twenge J. M. (1997). Changes in masculine and feminine traits over time: a meta-analysis. *Sex Roles* 36 305–325. 10.1007/BF02766650

[B75] Uekermann J. Kraemer M. Abdel-Hamid M. Schimmelmann B. G. Hebebrand J. Daum I. (2010). Social cognition in attention-deficit hyperactivity disorder (ADHD). *Neurosci. Biobehav. Rev.* 34 734–743. 10.1016/j.neubiorev.2009.10.009 19857516

[B76] Uzefovsky F. Shalev I. Israel S. Edelman S. Raz Y. Mankuta D. (2015). Oxytocin receptor and vasopressin receptor 1a genes are, respectively, associated with emotional and cognitive empathy. *Horm. Behav.* 67 60–65. 10.1016/j.yhbeh.2014.11.007 25476609

[B77] Uzefovsky F. Shalev I. Israel S. Edelman S. Raz Y. Perach-Barzilay N. (2014). The Dopamine D4 receptor gene shows a gender-sensitive association with cognitive empathy: evidence from two independent samples. *Emotion* 14 712–721. 10.1037/a0036555 24866520

[B78] van Anders S. M. Gray P. B. (2007). Hormones and human partnering. *Annu. Rev. Sex Res.* 18 60–93.

[B79] Volbrecht M. M. Lemery-Chalfant K. Aksan N. Zahn-Waxler C. Goldsmith H. H. (2007). Examining the familial link between positive affect and empathy development in the second year. *J. Genet. Psychol.* 168 105–130. 10.3200/GNTP.168.2.105-130 17936968 PMC3197271

[B80] Walker S. (2005). Gender differences in the relationship between young children’s peer-related social competence and individual differences in theory of mind. *J. Genet. Psychol.* 166 297–312. 10.3200/GNTP.166.3.297-312 16173673

[B81] Walter H. (2012). Social cognitive neuroscience of empathy: concepts, circuits, and genes. *Emotion Rev.* 4 9–17. 10.1177/1754073911421379

[B82] Wigginton J. Abecasis G. (2005). PEDSTATS: descriptive statistics, graphics and quality assessment for gene mapping data. *Bioinformatics* 21 3445–3447. 10.1093/bioinformatics/bti529 15947021

[B83] Wilmer J. B. Germine L. Chabris C. F. Chatterjee G. Williams M. Loken E. (2010). Human face recognition ability is specific and highly heritable. *Proc. Natl. Acad. Sci. U.S.A.* 107 5238–5241. 10.1073/pnas.0913053107 20176944 PMC2841913

[B84] Wu N. Li Z. Su Y. (2012). The association between oxytocin receptor gene polymorphism (OXTR) and trait empathy. *J. Affect. Disord.* 138 468–472. 10.1016/j.jad.2012.01.009 22357335

[B85] Wu N. Su Y. (2014). Oxytocin receptor gene relates to theory of mind and prosocial behavior in children. *J. Cogn. Dev.* 16 302–313. 10.1080/15248372.2013.858042

[B86] Yirmiya N. Sigman M. D. Kasari C. Mundy P. (1992). Empathy and cognition in high-functioning children with Autism. *Child Dev.* 63 150–160. 10.2307/1130909 1551323

[B87] Young L. J. Wang Z. (2004). The neurobiology of pair bonding. *Nat. Neurosci.* 7 1048–1054. 10.1038/nn1327 15452576

[B88] Zahn-Waxler C. Cole P. M. Barrett K. C. (1991). “Guilt and empathy: sex differences and implications for the development of depression,” in *The Development of Emotion Regulation and Dysregulation* eds Garber J. Dodge K. A. (Cambridge: Cambridge University Press) 243–272. 10.1017/CBO9780511663963.012

[B89] Zahn-Waxler C. Cummings E. M. Cooperman C. (1984). “Emotional development in childhood,” in *Annals of Child Development* Vol. 1 ed. Whitehurst C. (New York: JAI) 45–106

[B90] Zahn-Waxler C. Shirtcliff E. A. Marceau K. (2008). Disorders of childhood and adolescence: gender and psychopathology. *Annu. Rev. Clin. Psychol.* 4 275–303. 10.1146/annurev.clinpsy.3.022806.091358 18370618

[B91] Zhong S. Israel S. Shalev I. Xue H. Ebstein R. P. Chew S. H. (2010). Dopamine D4 receptor gene associated with fairness preference in ultimatum game. *PLoS ONE* 5:e13765 10.1371/journal.pone.0013765 PMC2972208 21072167

[B92] Zhu B. Chen C. Moyzis R. K. Dong Q. Chen C. He Q. (2011). Genetic variations in the dopamine system and facial expression recognition in healthy chinese college students. *Neuropsychobiology* 65 83–89. 10.1159/000329555 22222624

[B93] Zhu Q. Song Y. Hu S. Li X. Tian M. Zhen Z. (2010). Heritability of the specific cognitive ability of face perception. *Curr. Biol.* 20 137–142. 10.1016/j.cub.2009.11.067 20060296

